# The Progression of Acute Myeloid Leukemia from First Diagnosis to Chemoresistant Relapse: A Comparison of Proteomic and Phosphoproteomic Profiles

**DOI:** 10.3390/cancers12061466

**Published:** 2020-06-04

**Authors:** Elise Aasebø, Frode S. Berven, Randi Hovland, Stein Ove Døskeland, Øystein Bruserud, Frode Selheim, Maria Hernandez-Valladares

**Affiliations:** 1 Department of Clinical Science, University of Bergen, 5021 Bergen, Norway; Elise.Aasebo@uib.no (E.A.); Oystein.Bruserud@uib.no (Ø.B.); 2 The Department of Biomedicine, The Proteomics Unit at the University of Bergen (PROBE), University of Bergen, 5009 Bergen, Norway; Frode.Berven@uib.no (F.S.B.); Frode.Selheim@uib.no (F.S.); 3 The Department of Biomedicine, University of Bergen, 5009 Bergen, Norway; Stein.Doskeland@uib.no; 4 Department for Medical Genetics, Haukeland University Hospital, 5021 Bergen, Norway; Randi.Hovland@uib.no; 5 Department of Biological Sciences, University of Bergen, 5006 Bergen, Norway

**Keywords:** acute myeloid leukemia, proteome, phosphoproteome, markers, patient relapse, mass spectrometry, kinase, minimal residual disease, mitochondria, CDK, degranulation, secretion

## Abstract

Acute myeloid leukemia (AML) is an aggressive hematological malignancy. Nearly 50% of the patients who receive the most intensive treatment develop chemoresistant leukemia relapse. Although the leukemogenic events leading to relapse seem to differ between patients (i.e., regrowth from a clone detected at first diagnosis, progression from the original leukemic or preleukemic stem cells), a common characteristic of relapsed AML is increased chemoresistance. The aim of the present study was to investigate at the proteomic level whether leukemic cells from relapsed patients present overlapping molecular mechanisms that contribute to this chemoresistance. We used liquid chromatography–tandem mass spectrometry (LC–MS/MS) to compare the proteomic and phosphoproteomic profiles of AML cells derived from seven patients at the time of first diagnosis and at first relapse. At the time of first relapse, AML cells were characterized by increased levels of proteins important for various mitochondrial functions, such as mitochondrial ribosomal subunit proteins (MRPL21, MRPS37) and proteins for RNA processing (DHX37, RNA helicase; RPP40, ribonuclease P component), DNA repair (ERCC3, DNA repair factor IIH helicase; GTF2F1, general transcription factor), and cyclin-dependent kinase (CDK) activity. The levels of several cytoskeletal proteins (MYH14/MYL6/MYL12A, myosin chains; VCL, vinculin) as well as of proteins involved in vesicular trafficking/secretion and cell adhesion (ITGAX, integrin alpha-X; CD36, platelet glycoprotein 4; SLC2A3, solute carrier family 2) were decreased in relapsed cells. Our study introduces new targetable proteins that might direct therapeutic strategies to decrease chemoresistance in relapsed AML.

## 1. Introduction

Acute myeloid leukemia (AML) is an aggressive and heterogeneous hematological malignancy [1,2,3]. Although most patients with newly diagnosed AML achieve complete remission (CR) after intensive induction and consolidation therapy, more than half of them relapse within the next three years. Late relapse (i.e., after five years of remission) is very uncommon [4]. The overall long-term AML-free survival is, therefore, only about 50%, even for young and fit AML patients who tolerate intensive therapy [5].

Relapses derive from minimal residual disease (MRD) [6,7,8] developing from the original dominating clone, from a minor subclone, or through progression from the original leukemic or preleukemic stem cells [9,10,11]. Regardless of the origin, AML relapsed cells have an increased chemoresistance in common [5]. Relapsed AML patients are usually treated with salvage cytotoxic therapy, but current clinical trials test pathway-targeted agents and immunotherapy-based approaches [12]. Such approaches depend on the knowledge of potential therapeutic targets in relapsed AML cells. [13,14,15].

Two separate whole-exome sequencing (WES) studies performed with paired patient samples at diagnosis and at early or late relapse, respectively [16], showed that the patients presented different genetic events leading to relapse. Relapsed AML had usually acquired at least one relapse-specific mutation (e.g., in *FLT3*, *ASXL1* or *RUNX1*), whereas mutations in *NPM1* and in signaling genes (e.g., *NRAS*, *KIT, PTPN11)* were less frequent [17,18]. These observations further illustrated the heterogeneity of AML with regard to new leukemogenic events prior to the development of chemoresistant AML relapse.

Liquid chromatography–tandem mass spectrometry (LC–MS/MS) technology has been applied to the study of AML blasts at relapse compared to diagnosis in 10 patients [13,19]. The levels of several proteins involved in DNA repair were significantly increased, and signaling proteins such as KIT and STAT5 were significantly more phosphorylated in relapsed cells. Various molecules involved in survival, apoptosis, and metabolism were also modulated, but these observations were patient-specific. In a previous study, we utilized LC–MS/MS-based proteomics/phosphoproteomics and the super-SILAC (Stable Isotope Labeling with Amino acids in Cell culture) mix quantitation approach to compare the proteome and phosphoproteome of AML cells derived at the time of first diagnosis from patients who later became leukemia-free survivors to those of AML cells acquired from patients who relapsed after an initial intensive and potentially curative treatment [20]. In our present study, we used the same methodological approach to compare proteomic and phosphoproteomic profiles for paired samples derived at the first time of diagnosis and at later first relapse. All samples were prepared according to the same standardized guidelines, and the enrichment of AML cells was carefully controlled. The aim of the study was to investigate whether patients with leukemia relapse show similarities in their proteomic and phosphoproteomic profiles despite the previously described leukemogenic heterogeneity of AML relapse [9,10,11].

## 2. Results

### 2.1. Description of AML Patients and Patients’ Cells Included in the Study

**Figure 1:**
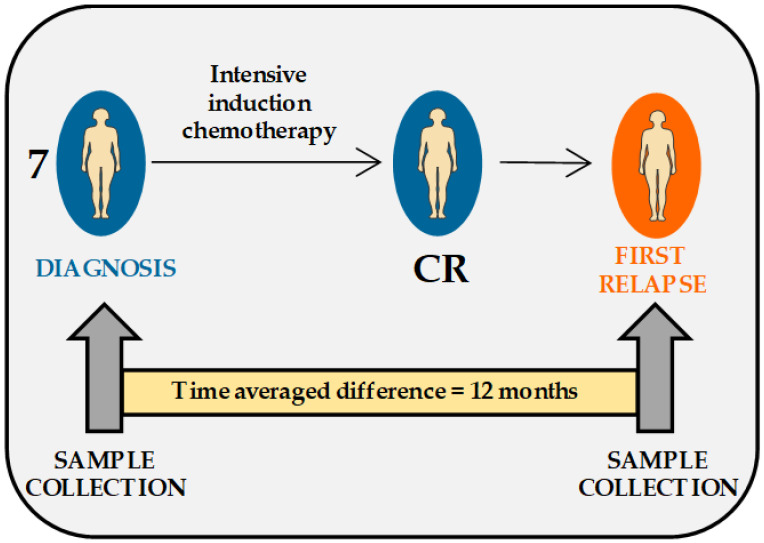
Overview of the matched diagnosis–first relapse patient cohort. The study included paired acute myeloid leukemia (AML) cell samples from seven patients, collected at the times of first diagnosis (DIAGNOSIS) and first relapse (FIRST RELAPSE). All patients received intensive induction chemotherapy and consolidation therapy and achieved complete remission (CR) (Table 1). All relapses occurred within three years after CR.

We investigated paired peripheral blood AML cell samples derived from seven patients at the time of first diagnosis (DIAGNOSIS samples) and at the time of first relapse (FIRST RELAPSE samples) (Figure 1).

To ensure that our patients were comparable, all samples included in the present study had to fulfill the following criteria: (i) a high percentage of AML cells among peripheral blood leukocytes both at the time of first diagnosis and at the time of first relapse; (ii) enriched AML cell populations including at least 90% of leukemic cells (documented both by microscopy and by flow cytometry) could thereby be prepared by highly standardized density gradient separation of viable cell suspensions [20]; (iii) all samples were thus derived from the same in vivo compartment, i.e., peripheral blood; and (iv) ex vivo handling of all blood samples was in accordance with the same standardized guidelines. We could thereby ensure a similar and high quality of all first diagnosis and first relapse samples included in the present study.

The first relapse occurred less than three years after the patients achieved CR. Clinical progression after CR is shown in Table 1 and described in Materials and Methods. Genetic analysis of paired DIAGNOSIS–FIRST RELAPSE samples was available for six patients and revealed several overlapping mutations. However, one patient acquired the mutation *FLT3*-ITD and another *RUNX1* + del (7q) at first relapse, while the relapsed AML cells of one patient no longer contained the abnormalities (*FLT3*-TDK, *IDH2, BCOR*) present at the time of first diagnosis (Table S1).

### 2.2. Mitochondrial Processing and Immune Responses at Relapse

The proteome profiles of AML patient cells derived from seven paired DIAGNOSIS–FIRST RELAPSE samples obtained using the super-SILAC mix approach allowed us to quantitatively determine the expression of 4132 proteins, against 3747 proteins using label-free (LF) quantitative proteomics (File S1), when including only proteins that yielded reliable FIRST RELAPSE/DIAGNOSIS fold change (FC) values for at least five out of the seven patients. The FC of the 3348 proteins quantified by the two quantification methods correlated well (Pearson *r* = 0.675; *p* < 0.0001; Figure S1).

The present DIAGNOSIS–FIRST RELAPSE proteome and phosphoproteome datasets, based on the super-SILAC mix results, were compared with those described in a larger cohort of AML samples collected at the time of first diagnosis from patients that either relapsed or had been AML-free for at least 5 years [20]. Few proteins and phosphorylation sites overlapped in both studies (Figure S2a,b), showing that the proteome and phosphoproteome changed considerably from the first diagnosis to the first relapse. We observed zinc finger proteins and phosphorylated RNA-binding proteins among the overlapped factors. The FC of expression and of phosphorylation had the same direction with regard to relapsed samples for all overlapping proteins and phosphorylated sites.

**Figure 2:**
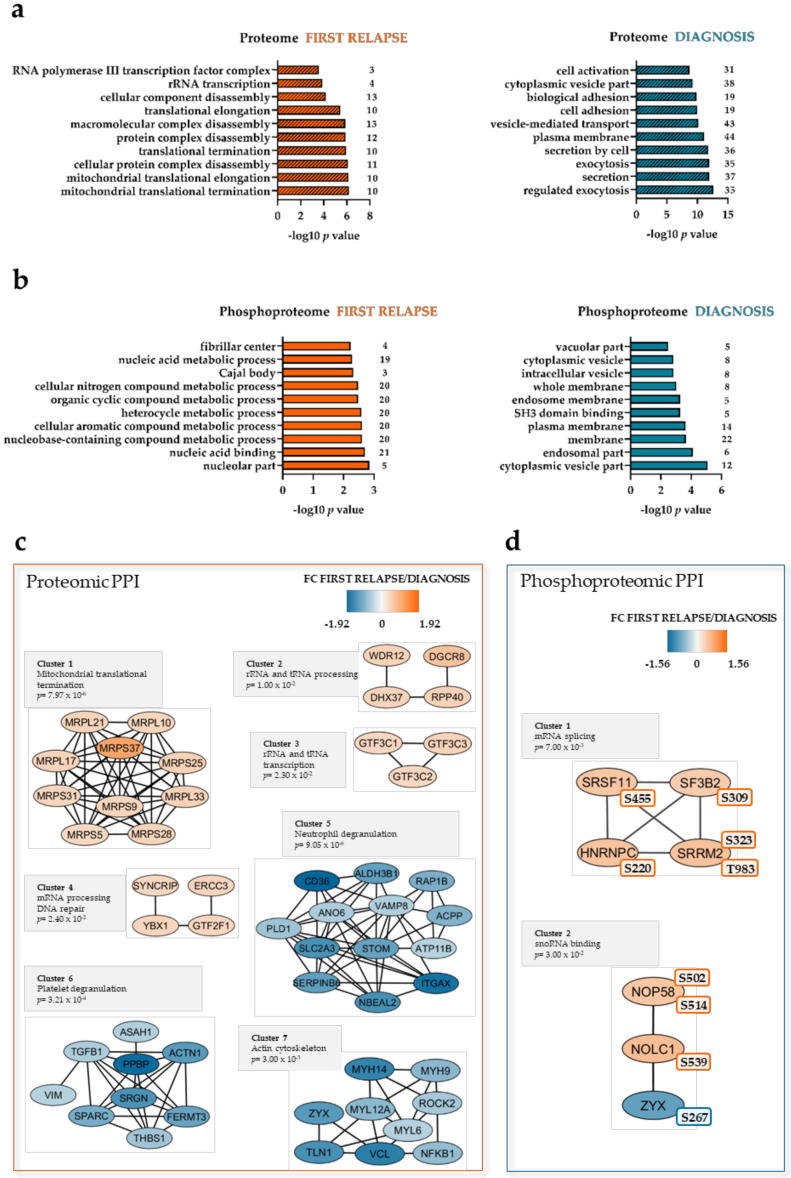
The AML proteome and phosphoproteome are enriched in mitochondrial ribosomal, RNA-processing, and DNA repair proteins at first relapse. Gene ontology (GO) analysis was conducted for the proteome (**a**) and phosphoproteome (**b**), separately. Top 10 enriched GO categories are shown on the y-axis of each bar plot. The -log_10_ *p* values and the number of proteins associated with these GO categories are shown on the X-axis. Dashed bars represent GO categories that are also significantly enriched with false discovery rate (FDR) < 0.05. (**c**,**d**) Networks of protein–protein interactions (PPI) based on STRING search of the proteomic and phosphoproteomic datasets and visualized in Cytoscape after ClusterONE analysis. Significance of high cohesiveness of protein and phosphoprotein networks is shown by the *p* value of a one-sided Mann–Whitney U test. Fold changes (FCs) of protein expression or phosphorylation according to the super-SILAC (Stable Isotope Labeling with Amino acids in Cell culture) mix method are color-coded; orange-colored proteins showed a higher expression or phosphorylation in the FIRST RELAPSE group, and Yale blue-colored proteins showed a higher expression or phosphorylation in the DIAGNOSIS group. The differentially regulated phosphorylation site(s) is shown next to each phosphoprotein.

Separate statistical analysis of the labeled proteomic and phosphoproteomic datasets indicated 168 differentially expressed proteins and 77 differentially regulated phosphorylation sites, respectively (Files S1 and S2). Mitochondrial proteins were significantly enriched at first relapse (Figure 2a, left part), such as 28S ribosomal proteins (Figure 2c, Cluster 1), mitochondrial respiratory chain complex proteins, and proteins involved in mitochondrial metabolism (Table S2). Nucleolar, nucleic acid-binding, and nucleic acid metabolism proteins were more phosphorylated at first relapse (Figure 2b, left part). Exocytosis and secretion as well as membrane and vesicle proteins were more abundant and phosphorylated, respectively, at diagnosis (Figure 2a,b, right part).

A protein–protein interaction (PPI) network of the differentially expressed proteins confirmed the increased abundance of mitochondrial, ribosomal, mRNA-, rRNA-, and tRNA-processing factors (Figure 2c, Cluster 1–4) at first relapse. RNA-splicing and -binding proteins were also more phosphorylated at first relapse (Figure 2d, Cluster 1,2).

Proteins with decreased cellular expression at the time of first relapse formed clusters of functional interaction (Figure 2c, Cluster 5–7) for neutrophil degranulation, platelet degranulation, and actin cytoskeleton (for details see Table S3). It should be noted (see also the Discussion section) that several of the proteins belonging to the neutrophil and platelet degranulation clusters are also important for the intracellular trafficking of endosomes or secretory vesicles [21] and that the cytoskeleton is important not only for endogenous cell function, but also for signaling initiated by cell surface adhesion molecules [22,23].

**Figure 3:**
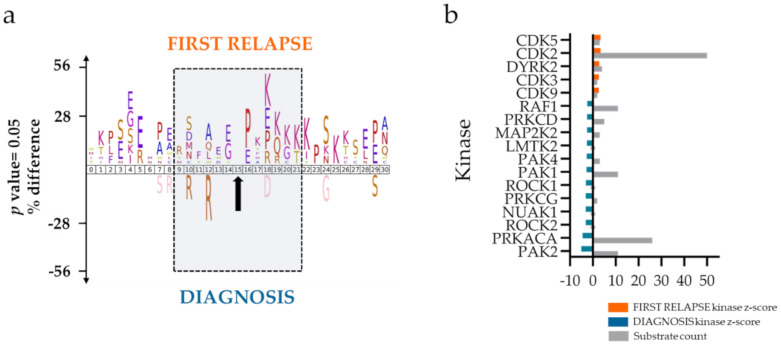
AML phosphoproteome in the FIRST RELAPSE group is enriched with cyclin-dependent kinases (CDKs) substrates. (**a**) Sequence motif analysis of the ± 31 amino acids flanking the differentially regulated phosphorylation sites (indicated with a black arrow) for the FIRST RELAPSE and DIAGNOSIS groups. (**b**) Kinase-substrate enrichment analysis (KSEA) of differentially regulated and unregulated phosphorylation sites. The kinase *z*-score (X axis) is the normalized score for each kinase (Y axis), weighted by the number of identified substrates.

The phosphoproteomes indicated, based on iceLogo [24] analysis of the amino acid sequences surrounding the differentially regulated phosphorylation sites, an increase of proline-directed and basophilic motifs in the FIRST RELAPSE group (Figure 3a), suggesting higher activating phosphorylation of mitogen-activated protein kinases (MAPKs) and cyclin-dependent kinases (CDKs), respectively. In the DIAGNOSIS group, an increase was noted for basophilic motifs located prior to the phosphorylation site characteristics of cAMP-dependent protein kinase catalytic subunit alpha (PRKACA) and protein kinase C family (PRKC).

A separate kinase-substrate enrichment analysis (KSEA) [25,26], using non-differentially regulated phosphorylation sites as background, supported an increased activity of CDKs in the FIRST RELAPSE group and of PRKACA, PRKC, and serine/threonine protein kinase PAK2 in the DIAGNOSIS group (Figure 3b).

Western blot analyses using lysates from six DIAGNOSIS–FIRST RELAPSE paired patient cells showed higher phosphorylation of CDK2 T160 in the FIRST RELAPSE samples (Figure S3), although the difference between the paired groups was at the limit of statistical significance (*p* = 0.0625). These findings correlate well with the kinase-substrate motif analyses showing higher relative abundance of phosphorylated CDK2 substrates in the FIRST RELAPSE group.

## 3. Discussion

Although stable remission can be induced by intensive antileukemic therapy for a majority of young and fit patients, about half of them relapse with chemoresistant AML [5]. AML cells from relapsed patients have been analyzed by whole-genome sequencing (WGS) or WES, but the protein and phosphoprotein patterns associated with AML relapse are less studied [16,27,28].

We have recently reported the proteomic and phosphoproteomic profiles of AML cells derived from patients at the first diagnosis before any antileukemic treatment [20]. The paired DIAGNOSIS–FIRST RELAPSE samples of the present study revealed significant alterations of the proteome and phosphoproteome between primary (diagnosis point) AML cells and the relapsed AML cells.

Our institution is responsible for the diagnostic evaluation and treatment of AML patients from a defined geographical area, including patients with relapsed disease. Our priority in the present study was not to include all patients during a defined time period, but to include all relapsed patients for whom it was possible to obtain AML cell samples that fulfilled our predefined quality criteria both at the time of first diagnosis and at the time of first relapse. Following the detection of AML relapse, antileukemic treatment has to be started for our patients, without additional delay [29]. Thus, for many of them it is not possible to prepare highly enriched AML cell populations that fulfill our quality criteria without extensive cell separation procedures that may influence the biological characteristics of the cells [30]. For these reasons, many of our relapsed patients could not be included in our present study. Despite this, it may be justified to say that our study is population-based, i.e., we included all relapsed patients from a defined geographical area and during a defined time, for whom AML cell samples fulfilling predefined quality criteria could be prepared both at the time of first diagnosis and the time of chemoresistant first relapse. Because of the full description of AML patients and their samples provided here, our present study should therefore be regarded as the first published complete report about a selection of high-quality samples that allowed reliable observations.

Analyses of GO categories showed higher expression of mitochondrial proteins in the FIRST RELAPSE group. This may be an adaption to the high level of lactate in advanced cancers and the leukemic bone marrow. Mitochondria may also contribute to drug resistance, since inhibitors of mitochondrial protein translation like tigecycline and tedizolid could restore drug sensitivity in human leukemic cell lines, suggesting a potential for the targeting of mitochondrial protein synthesis to treat AML [31,32,33,34]. RNA transcription and translation processes were also upregulated in the FIRST RELAPSE group. This observation is especially significant, since the levels of proteins associated with RNA transcription and translation were higher already at diagnosis for those patients that did relapse later [20]. It appears, therefore, that a high AML cell capacity for protein translation is associated with an increased relapse risk. A previous proteome and functional study showed that AML cells exposed in vitro to anthracycline responded with increased synthesis of survival proteins and became sensitized to death by protein synthesis inhibitors [35].

The AML proteome in the FIRST RELAPSE group had significantly decreased expression of proteins associated with the plasma membrane, exocytosis and secretory vesicles, as well as cytoskeletal proteins. A similar trend was noted when comparing the AML cell proteome at the first time of diagnosis from patients who later develop AML relapse and who become long-term AML-free survivors (i.e., at least a five-year survival) [20]. The NFκB transcription factor was also downregulated in the FIRST RELAPSE group. This protein appears to stimulate the transcription of several cytokines in AML cells, and its decreased expression may contribute to the observed lowered cytokine gene transcription in relapsed cells [36,37,38].

The downregulation of cytokines and cytoskeleton components contributing to intracellular endosomal trafficking and extracellular release of soluble mediators by secretory vesicles [39] indicate that relapsed or relapse-prone AML cells have decreased capacity of and probably need for normal intercellular signaling and contacts. This notion is supported by a previous study of the constitutive cytokine secretion profile during in vitro culture of AML patient cells [40,41], which showed that the low constitutive release of 41 soluble mediators was associated with unfavorable prognosis after intensive therapy. Thus, our findings are consistent with the hypothesis that chemoresistant AML cells are less dependent on the support from neighboring nonleukemic stromal cells mediated through the local cytokine network or through adhesion to the extracellular matrix or to neighboring stromal cells mediated by integrins or other adhesion molecules.

The alignment of amino acid sequences surrounding phosphorylation sites and KSEA showed enrichment of CDK substrates in the FIRST RELAPSE group. A similar kinase–substrate prediction scenario was observed in relapsed patients studied at diagnosis [20]. CDK inhibitors are currently investigated as a promising therapeutic strategy either in newly diagnosed or in relapsed AML cases [42,43,44]. The high CSK2 kinase activity of relapsed patients at diagnosis was no longer observed in chemoresistant cells after relapse. This may be explained if CSK2 has a role in inducing treatment-resistant clones but is dispensable for the survival of clones that already have become therapy-resistant. As observed for the relapse-free patients characterized at diagnosis [20], the phosphoproteome analysis in this study revealed activation of PRKA/C and PAK kinases in the DIAGNOSIS group.

A final point is whether the technologies used in the present study are suitable for routine clinical practice. The first question is then whether it is realistic to obtain sufficient leukemic cells for such analyses. In our experience, 20 μg of protein is needed for proteomic and at least 200 μg for phosphoproteomic analyses. We usually retrieve 5–15 × 10^6^ cells from each of our samples. Thus, it should be possible to achieve sufficient material for most patients. Secondly, will the results from such analyses be available before the antileukemic treatment is planned to start? In our experience, results from proteomic analyses can be available within three days, and phosphoproteomic results within four days, but this time can probably be shortened when the analyses are robotized. This is within the recommended time limit for the start of intensive antileukemic therapy [29]. Thirdly, this methodological approach should be possibly independent of the morphology or genetic abnormalities of AML cells. Finally, proteomic analyses should be carried out with regard to the prognostic evaluation of patients or as a guide for the selection of targeted therapies, whereas we would expect the analysis of AML-specific markers (e.g., genetic abnormalities) or single cell analyses to be more appropriate for the analysis of MRD [45,46]. Thus, although LC–MS/MS-based technologies could be suitable for clinical AML analysis, one should emphasize that additional clinical studies, standardization of the methods, and probably also development of faster bioinformatical tools will be required for a clinical implementation of such analyses.

## 4. Materials and Methods

The materials and methods of this study will be described briefly. Detailed workflows can be found in a previous publication of our group [20].

### 4.1. AML Patients and Sample Collection

Written informed consent was obtained from all patients in accordance with the Declaration of Helsinki. The use of human leukemia cells for the present study was approved by the Regional Ethics Committee (REK III Vest 2013-634). Primary AML cells were collected from the peripheral blood of patients with circulating leukemia blasts representing > 80% of circulating leukocytes, both at the first diagnosis (DIAGNOSIS) prior to treatment and at the time of first relapse (FIRST RELAPSE). The peripheral blood leukocyte counts are given in Table S1. Highly enriched (generally > 95%) AML cell populations were isolated by density gradient separation (Lymphoprep, Axis-Shield, Dundee, Scotland) [47]. This and other methodological strategies were described and discussed previously [20].

All patients received intensive induction and consolidation treatment leading to stable first CR, before relapse of the disease. They represented a consecutive (and thereby random) group of patients admitted to our hospital, fulfilling the following criteria: completed previous intensive antileukemic treatment, early relapse (i.e., within 27 months), and sufficient level of circulating AML cells for analysis at the time of both first diagnosis and first relapse. No samples were influenced by recent or ongoing anti-leukemic therapies.

All our patients received either intensive or supportive chemotherapy after diagnosis of first relapse. Six of them did not achieve a second CR and died within three months after diagnosis of first relapse. The remaining patient that achieved a second CR had allogeneic stem cell transplantation but died 10 months after first relapse (Table 1). The results of the mutational analysis of 54 genes frequently mutated in AML in the DIAGNOSIS and FIRST RELAPSE samples are shown in Table S1. The method for the genetic analyses has been described somewhere else [48].

### 4.2. Sample Preparation for LC–MS/MS

Cell lysates were prepared in 4% sodium dodecyl sulfate (SDS)/0.1 M Tris-HCl (pH 7.6). Proteomic and phosphoproteomic samples were spiked with a super-SILAC mix. Proteomic samples were also prepared for LF quantitation, which was used as internal validation for the labeled dataset [20]. All the samples were processed according to the filter-aided sample preparation (FASP) [49,50]. Only the super-SILAC spiked peptide samples were fractionated using styrenedivinylbenzene-reversed-phase sulfonate (SDB-RPS) plugs (Empore, 3M, St. Paul, MN, USA) [51]. Immobilized metal affinity chromatography (IMAC) was used for phosphopeptide enrichment [50].

### 4.3. Nanoflow LC–MS/MS and Data Analysis

Samples were run on a Q Exactive HF Orbitrap coupled to an Ultimate 3000 Rapid Separation LC system (Thermo Scientific, Waltham, MA, USA). The LC–MS/MS settings have been previously described [20]. Raw files were processed with MaxQuant software version 1.5.2.8 [52,53], and the Perseus 1.6.1.1 platform was used to analyze and visualize protein groups and phosphosites [20,54]. The LC–MS/MS raw files and MaxQuant output files were deposited in the ProteomeXchange Consortium via the PRIDE partner repository [55,56] with dataset identifier PXD018359. Proteins and phosphosites (localization probability > 0.75) with at least five FIRST RELAPSE/DIAGNOSIS FCs were selected for paired *t*-test and *Z*-statistics [57]. GO analysis was performed using a GO tool [58]. Venn diagrams were made with Biovenn [59]. The amino acid distribution surrounding the phosphosites was analyzed using iceLogo (*p* = 0.05) with the sequence windows obtained in the MaxQuant-generated phosphosite output file [24]. Unregulated phosphosites were used as a reference set. Kinase activity estimates were inferred by the KSEA App [20,25,26]. PPI networks were obtained by using the STRING database version 11 with interactions derived from experiments and databases at a high confidence score of 0.7 [60]. Networks were visualized using the Cytoscape platform version 3.7.2 [61]. The ClusterONE plugin was used to identify protein groups of high cohesiveness [62].

### 4.4. Western Blots

Western blots for six DIAGNOSIS–FIRST RELAPSE paired samples were performed as described earlier [20]. Eighteen µg of each sample was employed. The phospho-CDK2 (Thr160) antibody was purchased from Cell Signaling Technology (Leiden, The Netherlands) and used according to the manufacturer’s guidelines. Chemiluminescence was measured on a LAS-3000 imager (Fujifilm, Tokyo, Japan) after membrane incubation with SuperSignal West Atto Ultimate Sensitivity Substrate (Thermo Scientific). Band intensities for the phosphoprotein were determined by densitometry software Image J [63]. Band intensities of a protein spotted at approximately 17 kDa on Ponceau-stained membranes were used for normalization (Figure S3). Statistical analysis was performed using Wilcoxon matched-pairs singed rank test with GraphPad Prism (San Diego, CA, USA).

## 5. Conclusions

The comparison between paired samples from AML patients at first diagnosis and at first relapse showed that relapse was associated with significant increased expression of mitochondrial, ribosomal, and RNA-processing proteins. The phosphorylation of RNA-binding and -splicing proteins was also higher in AML cells after relapse. The relapsed AML cells had decreased expression of proteins involved in intracellular endosomal/secretory vesicle trafficking and cell adhesion.

Thus, although AML patients may be heterogeneous with regard to the primary mechanisms leading to relapse [9,10,11], the common features detected in the present study may be a result of converging effects secondary to the primary mechanisms behind the relapse. The findings suggest that relapsed AML cells have switched to a state with higher expression of mitochondrial proteins and activation of CDKs, as well as increased protein synthesis and RNA processing capacity.

In addition, specific changes for subsets or single patient samples were observed, particularly at the phosphoproteome level. These may represent specific individual changes at first relapse and might be useful to suggest personalized approaches to target disease recurrence.

Herein, we have shown that LC–MS/MS proteomics technology is also applicable to study AML relapse. Our recent studies support the use of this strategy at routine level to assist prognosis and treatment decisions, assuming that the necessary equipment, protocols, software, and expertise are available.

## Figures and Tables

**Table 1:** Clinical progression of AML patients from the time of first diagnosis until relapse.

Patient Code	Treatment at Diagnosis	Chemotherapy Cycles before CR	Time from CR1 to Relapse (Months)	Chemotherapy after Relapse	Response to Intensive Chemotherapy	Survival after Diagnosed First Relapse (Months)
PX1	ICT/CCT	2	8	ICT	No CR2, death from chemoresistant disease	3
PX2	ICT/CCT	1	16	ST	No CR2	1
PX3	ICT/CCT	1	27	ICT	Allogeneic retransplantation in CR2, death due to chemoresistant second relapse	10
PX4	ICT/CCT	1	13	ST	No CR2	2
PX5	ICT/CCT	1	5	ICT	No CR2, death due to infection during neutropenia	1
PX6	ICT/CCT	1	3	ICT	No CR2, death from chemoresistant disease	2
PX7	ICT/CCT	2	2	ICT	No CR2	3

CR: 1, first remission; 2, second remission; ICT: induction chemotherapy; CCT: consolidation chemotherapy; ST: supportive therapy.

## Supplementary Materials

The following are available online at https://www.mdpi.com/2072-6694/12/6/1466/s1. Figure S1: The median FIRST RELAPSE/DIAGNOSIS fold change (FC) of proteins correlate in the super-SILAC (SS) and label-free (LF) datasets, Figure S2: Comparison of regulated factors between the RELAPSE vs. REL_FREE and FIRST RELAPSE vs. DIAGNOSIS cohorts, Figure S3: Ponceau-stained membranes and Western blots, Table S1: Mutational and cytogenetic characterization of the AML patients at diagnosis (D) and at first relapse (R), Table S2: List of upregulated mitochondrial proteins at first relapse identified by Gene Ontology (GO) enrichment, Table S3: A more detailed description of the proteins showing decreased levels in relapse patients and included in the neutrophil degranulation, platelet degranulation and actin cytoskeleton clusters of Figure 2c of the main text, File S1: Proteomics data FIRST RELAPSE vs. DIAGNOSIS cohort (SS-mix and LF datasets), File S2: Phosphoproteomics data FIRST RELAPSE vs. DIAGNOSIS cohort (SS-mix dataset).

## References

[1] Ferrara F., Schiffer C.A. (2013). Acute myeloid leukaemia in adults. Lancet.

[2] Arber D.A., Orazi A., Hasserjian R., Thiele J., Borowitz M.J., Le Beau M.M., Bloomfield C.D., Cazzola M., Vardiman J.W. (2016). The 2016 revision to the World Health Organization classification of myeloid neoplasms and acute leukemia. Blood.

[3] Versluis J., Cornelissen J.J., Craddock C., Sanz M.A., Canaani J., Nagler A., Carreras E., Dufour C., Mohty M., Kroger N. (2019). Acute Myeloid Leukemia in Adults. The EBMT Handbook: Hematopoietic Stem Cell Transplantation and Cellular Therapies.

[4] Mariani S., Trisolini S.M., Minotti C., Breccia M., Cartoni C., De Propris M.S., Loglisci G., Latagliata R., Limongi M.Z., Testi A.M. (2020). Very late acute myeloid leukemia relapse: Clinical features, treatment and outcome. Leuk. Lymphoma.

[5] Dohner H., Estey E., Grimwade D., Amadori S., Appelbaum F.R., Buchner T., Dombret H., Ebert B.L., Fenaux P., Larson R.A. (2017). Diagnosis and management of AML in adults: 2017 ELN recommendations from an international expert panel. Blood.

[6] Schuurhuis G.J., Heuser M., Freeman S., Bene M.C., Buccisano F., Cloos J., Grimwade D., Haferlach T., Hills R.K., Hourigan C.S. (2018). Minimal/measurable residual disease in AML: A consensus document from the European LeukemiaNet MRD Working Party. Blood.

[7] Paietta E. (2018). Consensus on MRD in AML?. Blood.

[8] Rowe J.M. (2018). Progress and predictions: AML in 2018. Best Pract. Res. Clin. Haematol..

[9] Corces M.R., Chang H.Y., Majeti R. (2017). Preleukemic Hematopoietic Stem Cells in Human Acute Myeloid Leukemia. Front. Oncol..

[10] Ediriwickrema A., Aleshin A., Reiter J.G., Corces M.R., Kohnke T., Stafford M., Liedtke M., Medeiros B.C., Majeti R. (2020). Single-cell mutational profiling enhances the clinical evaluation of AML MRD. Blood Adv..

[11] Horibata S., Alyateem G., DeStefano C.B., Gottesman M.M. (2020). The Evolving AML Genomic Landscape: Therapeutic Implications. Curr. Cancer Drug Targets.

[12] Ramos N.R., Mo C.C., Karp J.E., Hourigan C.S. (2015). Current Approaches in the Treatment of Relapsed and Refractory Acute Myeloid Leukemia. J. Clin. Med..

[13] Britton D.J., Wilkes E., Casado P., Rajeeve V., Fitzgibbon J., Gribben J., Cutillas P.R. (2016). Proteomic Analysis Directs Effective Drug Selection in Relapsed AML By Quantifying Drug Targets. Blood.

[14] Ball B., Stein E.M. (2019). Which are the most promising targets for minimal residual disease-directed therapy in acute myeloid leukemia prior to allogeneic stem cell transplant?. Haematologica.

[15] Schraw J.M., Junco J.J., Brown A.L., Scheurer M.E., Rabin K.R., Lupo P.J. (2019). Metabolomic profiling identifies pathways associated with minimal residual disease in childhood acute lymphoblastic leukaemia. Ebiomedicine.

[16] Yilmaz M., Wang F., Loghavi S., Bueso-Ramos C., Gumbs C., Little L., Song X.Z., Zhang J.H., Kadia T., Borthakur G. (2019). Late relapse in acute myeloid leukemia (AML): Clonal evolution or therapy-related leukemia?. Blood Cancer J..

[17] Cocciardi S., Dolnik A., Kapp-Schwoerer S., Rucker F.G., Lux S., Blatte T.J., Skambraks S., Kronke J., Heidel F.H., Schnoder T.M. (2019). Clonal evolution patterns in acute myeloid leukemia with NPM1 mutation. Nat. Commun..

[18] Hollein A., Meggendorfer M., Dicker F., Jeromin S., Nadarajah N., Kern W., Haferlach C., Haferlach T. (2018). NPM1 mutated AML can relapse with wild-type NPM1: Persistent clonal hematopoiesis can drive relapse. Blood Adv..

[19] Cutillas P.R. (2020). Personal communication.

[20] Aasebo E., Berven F.S., Bartaula-Brevik S., Stokowy T., Hovland R., Vaudel M., Doskeland S.O., McCormack E., Batth T.S., Olsen J.V. (2020). Proteome and Phosphoproteome Changes Associated with Prognosis in Acute Myeloid Leukemia. Cancers.

[21] Ramadass M., Catz S.D. (2016). Molecular mechanisms regulating secretory organelles and endosomes in neutrophils and their implications for inflammation. Immunol. Rev..

[22] Kadry Y.A., Calderwood D.A. (2020). Chapter 22: Structural and signaling functions of integrins. Biochim. Biophys. Acta Biomembr..

[23] Michael M., Parsons M. (2020). New perspectives on integrin-dependent adhesions. Curr. Opin. Cell Biol..

[24] Colaert N., Helsens K., Martens L., Vandekerckhove J., Gevaert K. (2009). Improved visualization of protein consensus sequences by iceLogo. Nat. Methods.

[25] Casado P., Rodriguez-Prados J.C., Cosulich S.C., Guichard S., Vanhaesebroeck B., Joel S., Cutillas P.R. (2013). Kinase-Substrate Enrichment Analysis Provides Insights into the Heterogeneity of Signaling Pathway Activation in Leukemia Cells. Sci. Signal..

[26] Wiredja D.D., Koyuturk M., Chance M.R. (2017). The KSEA App: A web-based tool for kinase activity inference from quantitative phosphoproteomics. Bioinformatics.

[27] Ding L., Ley T.J., Larson D.E., Miller C.A., Koboldt D.C., Welch J.S., Ritchey J.K., Young M.A., Lamprecht T., McLellan M.D. (2012). Clonal evolution in relapsed acute myeloid leukaemia revealed by whole-genome sequencing. Nature.

[28] Wang Y.W., Tsai C.H., Lin C.C., Tien F.M., Chen Y.W., Lin H.Y., Yao M., Lin Y.C., Lin C.T., Cheng C.L. (2020). Cytogenetics and mutations could predict outcome in relapsed and refractory acute myeloid leukemia patients receiving BCL-2 inhibitor venetoclax. Ann. Hematol..

[29] Sekeres M.A., Elson P., Kalaycio M.E., Advani A.S., Copelan E.A., Faderl S., Kantarjian H.M., Estey E. (2009). Time from diagnosis to treatment initiation predicts survival in younger, but not older, acute myeloid leukemia patients. Blood.

[30] Bruserud O., Gjertsen B.T., Foss B., Huang T.S. (2001). New strategies in the treatment of acute myelogenous leukemia (AML): In Vitro culture of aml cells—The present use in experimental studies and the possible importance for future therapeutic approaches. Stem Cells.

[31] Skrtic M., Sriskanthadevan S., Jhas B., Gebbia M., Wang X., Wang Z., Hurren R., Jitkova Y., Gronda M., Maclean N. (2011). Inhibition of mitochondrial translation as a therapeutic strategy for human acute myeloid leukemia. Cancer Cell.

[32] Schimmer A.D., Skrtic M. (2012). Therapeutic potential of mitochondrial translation inhibition for treatment of acute myeloid leukemia. Expert Rev. Hematol..

[33] Sharon D., Cathelin S., Subedi A., Williams R., Benicio M., Ketela T., Chan S.M. (2017). Targeting Mitochondrial Translation Overcomes Venetoclax Resistance in Acute Myeloid Leukemia (AML) through Activation of the Integrated Stress Response. Blood.

[34] Sharon D., Cathelin S., Mirali S., Di Trani J.M., Yanofsky D.J., Keon K.A., Rubinstein J.L., Schimmer A.D., Ketela T., Chan S.M. (2019). Inhibition of mitochondrial translation overcomes venetoclax resistance in AML through activation of the integrated stress response. Sci. Transl. Med..

[35] Gausdal G., Gjertsen B.T., McCormack E., Damme P., Hovland R., Krakstad C., Bruserud O., Gevaert K., Vandekerckhove J., Doskeland S.O. (2008). Abolition of stress-induced protein synthesis sensitizes leukemia cells to anthracycline-induced death. Blood.

[36] Bruserud O., Ryningen A., Olsnes A.M., Stordrange L., Oyan A.M., Kalland K.H., Gjertsen B.T. (2007). Subclassification of patients with acute myelogenous leukemia based on chemokine responsiveness and constitutive chemokine release by their leukemic cells. Haematologica.

[37] Reikvam H., Hatfield K.J., Lassalle P., Kittang A.O., Ersvaer E., Bruserud O. (2010). Targeting the angiopoietin (Ang)/Tie-2 pathway in the crosstalk between acute myeloid leukaemia and endothelial cells: Studies of Tie-2 blocking antibodies, exogenous Ang-2 and inhibition of constitutive agonistic Ang-1 release. Expert Opin. Investig. Drugs.

[38] Reikvam H., Hatfield K.J., Oyan A.M., Kalland K.H., Kittang A.O., Bruserud O. (2010). Primary human acute myelogenous leukemia cells release matrix metalloproteases and their inhibitors: Release profile and pharmacological modulation. Eur. J. Haematol..

[39] Li P., Bademosi A.T., Luo J., Meunier F.A. (2018). Actin Remodeling in Regulated Exocytosis: Toward a Mesoscopic View. Trends Cell Biol..

[40] Brenner A.K., Reikvam H., Bruserud O. (2016). A Subset of Patients with Acute Myeloid Leukemia Has Leukemia Cells Characterized by Chemokine Responsiveness and Altered Expression of Transcriptional as well as Angiogenic Regulators. Front. Immunol..

[41] Honnemyr M., Bruserud O., Brenner A.K. (2017). The constitutive protease release by primary human acute myeloid leukemia cells. J. Cancer Res. Clin. Oncol..

[42] Lee D.J., Zeidner J.F. (2019). Cyclin-dependent kinase (CDK) 9 and 4/6 inhibitors in acute myeloid leukemia (AML): A promising therapeutic approach. Expert Opin. Investig. Drugs.

[43] Xie S., Jiang H., Zhai X.W., Wei F., Wang S.D., Ding J., Chen Y. (2016). Antitumor action of CDK inhibitor LS-007 as a single agent and in combination with ABT-199 against human acute leukemia cells. Acta Pharmacol. Sin..

[44] Li K.L., Bray S.C., Iarossi D., Adams J., Zhong L.J., Noll B., Rahaman M.H., Richmond J., To L.B., Lewis I.D. (2015). Investigation of a Novel Cyclin-Dependent-Kinase (CDK) Inhibitor Cdki-73 As an Effective Treatment Option for MLL-AML. Blood.

[45] Jongen-Lavrencic M., Grob T., Hanekamp D., Kavelaars F.G., Al Hinai A., Zeilemaker A., Erpelinck-Verschueren C.A.J., Gradowska P.L., Meijer R., Cloos J. (2018). Molecular Minimal Residual Disease in Acute Myeloid Leukemia. N. Engl. J. Med..

[46] Ossenkoppele G., Schuurhuis G.J. (2016). MRD in AML: Does it already guide therapy decision-making?. Hematol. Am. Soc. Hematol. Educ. Program..

[47] Hatfield K.J., Hovland R., Øyan A.M., Kalland K.H., Ryningen A., Gjertsen B.T., Bruserud Ø. (2008). Release of angiopoietin-1 by primary human acute myelogenous leukemia cells is associated with mutations of nucleophosmin, increased by bone marrow stromal cells and possibly antagonized by high systemic angiopoietin-2 levels. Leukemia.

[48] Reikvam H., Hovland R., Forthun R.B., Erdal S., Gjertsen B.T., Fredly H., Bruserud O. (2017). Disease-stabilizing treatment based on all-trans retinoic acid and valproic acid in acute myeloid leukemia-identification of responders by gene expression profiling of pretreatment leukemic cells. BMC Cancer.

[49] Wisniewski J.R., Zougman A., Nagaraj N., Mann M. (2009). Universal sample preparation method for proteome analysis. Nat. Methods.

[50] Hernandez-Valladares M., Aasebø E., Mjaavatten O., Vaudel M., Bruserud Ø., Berven F., Selheim F. (2016). Reliable FASP-based procedures for optimal quantitative proteomic and phosphoproteomic analysis on samples from acute myeloid leukemia patients. Biol. Proced. Online.

[51] Kulak N.A., Pichler G., Paron I., Nagaraj N., Mann M. (2014). Minimal, encapsulated proteomic-sample processing applied to copy-number estimation in eukaryotic cells. Nat. Methods.

[52] Cox J., Mann M. (2008). MaxQuant enables high peptide identification rates, individualized p.p.b.-range mass accuracies and proteome-wide protein quantification. Nat. Biotechnol..

[53] Cox J., Matic I., Hilger M., Nagaraj N., Selbach M., Olsen J.V., Mann M. (2009). A practical guide to the MaxQuant computational platform for SILAC-based quantitative proteomics. Nat. Protoc..

[54] Tyanova S., Temu T., Sinitcyn P., Carlson A., Hein M.Y., Geiger T., Mann M., Cox J. (2016). The Perseus computational platform for comprehensive analysis of (prote)omics data. Nat. Methods.

[55] Deutsch E.W., Csordas A., Sun Z., Jarnuczak A., Perez-Riverol Y., Ternent T., Campbell D.S., Bernal-Llinares M., Okuda S., Kawano S. (2017). The ProteomeXchange consortium in 2017: Supporting the cultural change in proteomics public data deposition. Nucleic Acids Res..

[56] Perez-Riverol Y., Csordas A., Bai J., Bernal-Llinares M., Hewapathirana S., Kundu D.J., Inuganti A., Griss J., Mayer G., Eisenacher M. (2019). The PRIDE database and related tools and resources in 2019: Improving support for quantification data. Nucleic Acids Res..

[57] Arntzen M.Ø., Koehler C.J., Barsnes H., Berven F.S., Treumann A., Thiede B. (2011). IsobariQ: Software for isobaric quantitative proteomics using IPTL, iTRAQ, and TMT. J. Proteome Res..

[58] Scholz C., Lyon D., Refsgaard J.C., Jensen L.J., Choudhary C., Weinert B.T. (2015). Avoiding abundance bias in the functional annotation of post-translationally modified proteins. Nat. Methods.

[59] Hulsen T., de Vlieg J., Alkema W. (2008). BioVenn—A web application for the comparison and visualization of biological lists using area-proportional Venn diagrams. BMC Genom..

[60] Szklarczyk D., Morris J.H., Cook H., Kuhn M., Wyder S., Simonovic M., Santos A., Doncheva N.T., Roth A., Bork P. (2017). The STRING database in 2017: Quality-controlled protein-protein association networks, made broadly accessible. Nucleic Acids Res..

[61] Shannon P., Markiel A., Ozier O., Baliga N.S., Wang J.T., Ramage D., Amin N., Schwikowski B., Ideker T. (2003). Cytoscape: A software environment for integrated models of biomolecular interaction networks. Genome Res..

[62] Nepusz T., Yu H., Paccanaro A. (2012). Detecting overlapping protein complexes in protein-protein interaction networks. Nat. Methods.

[63] Schneider C.A., Rasband W.S., Eliceiri K.W. (2012). NIH Image to ImageJ: 25 years of image analysis. Nat. Methods.

