# Supplementary material for: The Progression of Acute Myeloid Leukemia from First Diagnosis to Chemoresistant Relapse: A Comparison of Proteomic and Phosphoproteomic Profiles
Source: Cancers (Basel). 2020 Jun 4;12(6):1466. doi: 10.3390/cancers12061466 (PMC7352627; doi:10.3390/cancers12061466)
Supplement: Supplementary file 1 [file cancers-12-01466-s001.zip › cancers-821562 - supplementary material/cancers-821562 - supplementary material.pdf]

Supplementary Material

The Progression of Acute Myeloid Leukemia from First Diagnosis to Chemoresistant Relapse: A Comparison of Proteomic and Phosphoproteomic Profiles

Elise Aasebø, Frode S. Berven, Randi Hovland, Stein Ove Døskeland, Øystein Bruserud, Frode Selheim and Maria Hernandez-Valladares

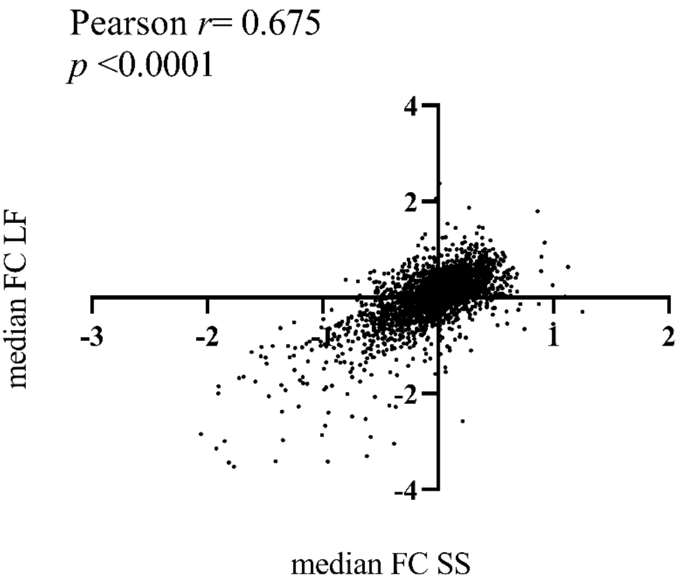

Figure S1. The median FIRST RELAPSE/DIAGNOSIS fold change (FC) of proteins correlate in the super-SILAC (SS) and label-free (LF) datasets. Pearson *r* value and correlation plot of 3348 protein FCs obtained from label-free and super-SILAC experiments using seven paired patient samples.

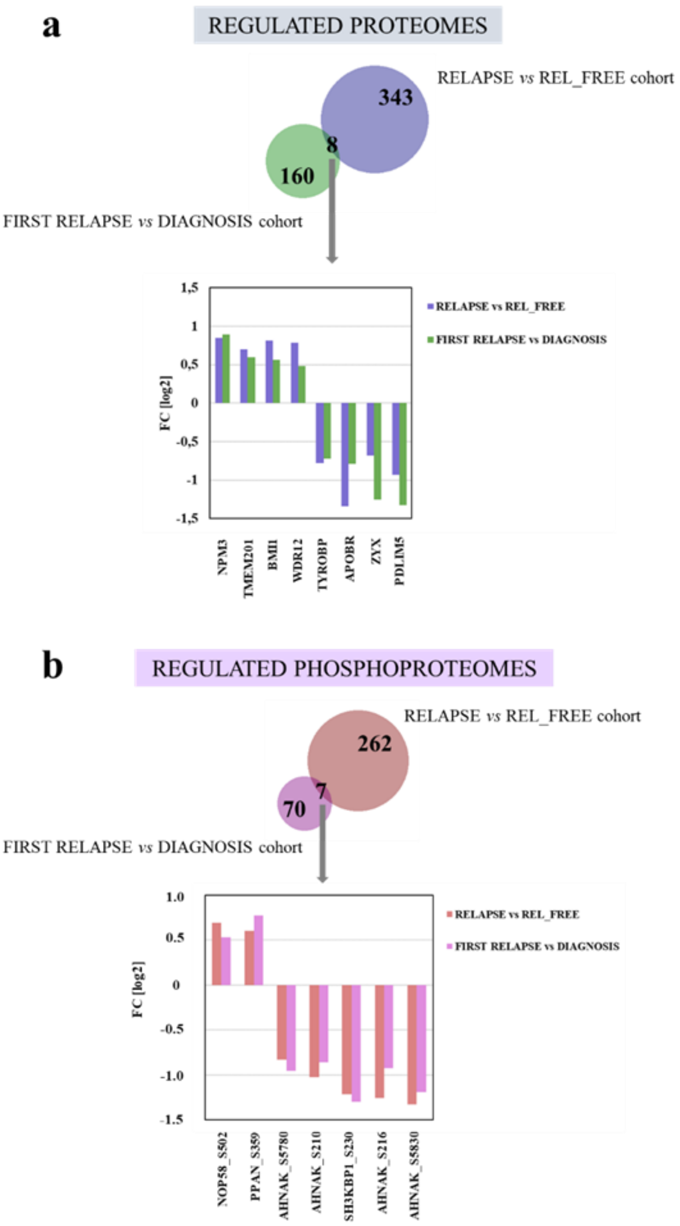

Figure S2. Comparison of regulated factors between the RELAPSE vs. REL_FREE and FIRST RELAPSE vs. DIAGNOSIS cohorts. (**a**) Overlap of eight differentially regulated proteins and their fold changes (FCs) measured in both cohorts. These FCs were not significantly different ($p = 0.3592$); (**b**) Overlap of seven differentially regulated phosphorylation sites and their FCs measured in both cohorts. These FCs were not significantly different ($p = 0.4019$). (The RELAPSE vs. REL_FREE proteome and phosphoproteome datasets were obtained from our previous study on acute myeloid leukemia (AML) patient samples collected at time of first diagnosis [1]).

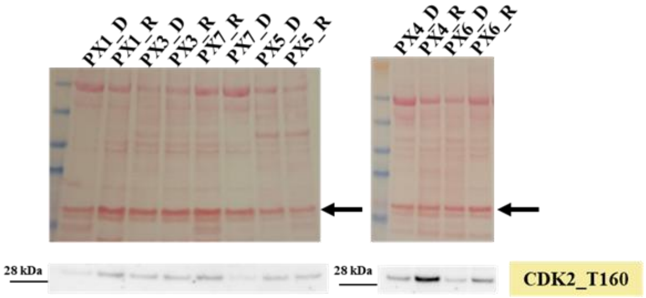

	PX1_D	PX1_R	PX3_D	PX3_R	PX7_R	PX7_D	PX5_D	PX5_R	PX4_D	PX4_R	PX6_D	PX6_R
Control band	4884,97	9221,05	7429,83	7604,21	8456,45	6806,57	5233,72	5116,08	5443,67	6545,82	5832,06	6486,94
CDK2_T160	625,31	2713,67	1470,82	1855,36	3469,55	712,06	1921,82	1860,41	2195,11	10000,65	1054,41	3530,65
ratio CDK2_T160/Control band	0,13	0,29	0,20	0,24	0,41	0,10	0,37	0,36	0,40	1,53	0,18	0,54

Wilcoxon matched-pairs signed rank test
p value 0.0625

Figure S3. Ponceau-stained membranes and Western blots. Ponceau-stained membranes, used as loading controls (the arrows mark the 17kDa-band intensity taking for normalization purposes), and Western blots of sample lysates from primary cells of six DIAGNOSIS-FIRST RELAPSE paired cases. Western blots were not replicated. Band intensities of T160-phosphorylated CDK2 were normalized before statistical analysis (see the table for intensity ratios). DIAGNOSIS-FIRST RELAPSE paired groups were compared using the Wilcoxon matched-pairs signed rank test. (PX7 paired samples appear swapped on the gel due to a swapped tube location on the loading rack).

Table S1. Mutational and cytogenetic characterization of the AML patients at diagnosis (D) and at first relapse (R).

					NPM1	SIGNALING						TUMOR SUPRESSORS				DNA METHYLATION					MYELOID TF		CHROMATIN MODIFICATION				COHESIN		SPLICEOSOME/TR			OTHERS		
Patient code	WBC count (10^9/L)	Clinical progression	Sample collection point	Time to relapse (months after diagnosis)	*NPM1* Ins	*FLT3*-ITD	*FLT3*-TKD	*NRAS*	*HRAS*	*KIT*	*PTPN11*	*IKZF1*	*CDKN2A*	*PHF6*	*WT1*	*DNMT3A*	*TET2*	*IDH1*	*IDH2*	*KMT2A*	*RUNX1*	*CEPBA*	*GATA2*	*ASXL1*	*EZH2*	*KDM6A*	*RAD21*	*STAG2*	*BCORL1*	*BCOR*	*SRSF2*	*CALR*	*CFR3R*	Cytogenetics
PX1_D	19	CR1 - RELAPSE	DIAGNOSIS	12	W288fs	97 bp																												46,XY
PX1_R	21		RELAPSE		W288fs	165 bp									V379fs*																			46,XY
PX2_D	10	CR1 - RELAPSE	DIAGNOSIS	16	W288fs												A1341E																	46,XX
PX2_R	12		RELAPSE		W288fs												A1341E																	
PX3_D	27	CR1 - RELAPSE	DIAGNOSIS	27	W288fs			G13R								G699S			R140Q															46,XY
PX3_R	28		RELAPSE		W288fs	30, 39, 6, 18, 12 bp		<u>G12D</u>								G699S			R140Q															46,XY
PX4_D	21	CR1 - RELAPSE	DIAGNOSIS	13																														48,XX,+6,+21,inc[4]/46, XX[17]
PX4_R	87		RELAPSE																															47~48,XX,del(2)(q12q14)(q22q24)[7]+6[13], +21[4][cp13]/46,XX[5]
PX5_D	14	CR1 - RELAPSE	DIAGNOSIS	6		39, 24, 108 bp							A148V			R882H																		46,XY,del(9)(q13q33)[18]
PX5_R	20		RELAPSE			39, 15, 108, 24 bp							A148V			R882H																		46,XY,del(9)(q13q33)[10]
PX6_D	14	CR1 - RELAPSE	DIAGNOSIS	6	W288fs	15, 54, 150 bp	D835Y					P142T				R882H			R140Q											A25fs, I1212T				46,XY
PX6_R	51		RELAPSE		W288fs							P142T				R882H																		
PX7_D	48	CR1 - RELAPSE	DIAGNOSIS	4			D835V							G186fs, G275D				R132H		PTD								M336fs						46,XX,dup(11)(q23.3q23.3)
PX7_R	29		RELAPSE				D835V							G186fs				R132H		PTD	R201Q							M336fs						46,XX,del(7)(q33), dup(11)(q23.3q23.3)[5] /46,XX[15]
					NM_002520	NM_004119	NM_004119	NM_002524				NM_001220773	NM_000077	NM_001015877	NM_024426	NM_022552	NM_001127208	NM_005896	NM_002168	NM_001197104.2	NM_001754							NM_001042751		NM_001123383				

Gene cell colors indicate: light yellow = wild type; light green = mutation; grey = not determined. Grey colored cells on the Cytogenetics column indicate not determined data. TF/TR: transcription factors/repressors; WBC: white blood cells; CR: complete remission; NPM1 Ins: a 4 bp-insertion/duplication; ITD: internal tandem duplication; TDK: tyrosine kinase domain; PTD: partial tandem duplication. The underlined mutation means that variant frequency was < 0.04. Mutation accession numbers are shown on the neath of mutation columns. (The patient characterization at diagnosis has been previously published [1]. Here it is shown to compare the characterization of the patients at diagnosis and at first relapse time points).

Table S2. List of upregulated mitochondrial proteins at first relapse identified by Gene Ontology (GO) enrichment.

UniProtKB AC/ID	Protein names	Gene names
O75394	39S ribosomal protein L33, mitochondrial	MRPL33 C2orf1
P82663	28S ribosomal protein S25, mitochondrial	MRPS25 RPMS25
P82675	28S ribosomal protein S5, mitochondrial	MRPS5
P82933	28S ribosomal protein S9, mitochondrial	MRPS9 RPMS9
Q7Z2W9	39S ribosomal protein L21, mitochondrial	MRPL21
Q7Z7H8	39S ribosomal protein L10, mitochondrial	MRPL10 MRPL8 RPML8
Q92665	28S ribosomal protein S31, mitochondrial	MRPS31 IMOGN38
Q96BP2	Coiled-coil-helix-coiled-coil-helix domain-containing protein 1	CHCHD1 C10orf34 MRPS37
Q9NRX2	39S ribosomal protein L17, mitochondrial	MRPL17 LIP2
Q9Y2Q9	28S ribosomal protein S28, mitochondrial	MRPS28 MRPS35 HSPC007
Q6PI78	Transmembrane protein 65	TMEM65
Q9BQ95	Evolutionarily conserved signaling intermediate in Toll pathway, mitochondrial	ECSIT
Q9NPL8	Complex I assembly factor TIMMDC1, mitochondrial	TIMMDC1 C3orf1 UNQ247/PRO284
Q9Y5J9	Mitochondrial import inner membrane translocase subunit Tim8 B	TIMM8B DDP2 DDPL TIM8B

Table S3. A more detailed description of the proteins showing decreased levels in relapse patients and included in the neutrophil degranulation, platelet degranulation and actin cytoskeleton clusters of Figure 2c of the main text. The information is based on the Gene database and selected references from PubMed.

Protein	Name and Description	Keywords
	**Neutrophil Degranulation (13 Proteins)**	
**ALDH3B1**	*Aldehyde dehydrogenase 3 family member B1.* Aldehyde dehydrogenases may play a role in the oxidation of lipid-derived aldehydes (i.e. long-chain fatty aldehydes) and may play a role in protection from oxidative stress. The enzyme is localized to the plasma membrane [2].	Plasma membrane<br>Detoxification
**CD36**	*CD36 molecule*. The protein encoded by this gene is the fourth major glycoprotein of the platelet surface and serves as a receptor for thrombospondin in platelets and various cell lines. This protein may have important functions as a cell adhesion molecule. It binds to collagen, thrombospondin, anionic phospholipids and oxidized LDL.	Cell adhesion
**RAP1B**	*RAP1B, member of RAS oncogene family.* This gene encodes a member of the RAS-like small GTP-binding protein superfamily that regulates integrin-mediated cell signaling. GTP-Rap1 seems involved in extracellular mediator secretion.	Secretion
**PLD1**	*Phospholipase D1.* This phosphatidylcholine-specific phospholipase may play a role in signal transduction and subcellular trafficking [3].	Subcellular trafficking
**ANO6**	*Anoctamin 6.* This transmembrane protein is a component for the calcium-dependent exposure of phosphatidylserine on the cell surface. It is a regulator of extracellular electrolyte secretion and the extracellular ADAM protease network [4,5].	Protease<br>Cytokine release-activation<br>Il6 family activity
**VAMP8**	*Vesicle associated membrane protein 8.* This integral membrane protein belongs to the synaptobrevin/vesicle-associated membrane protein subfamily of soluble N-ethylmaleimide-sensitive factor attachment protein receptors (SNAREs). The encoded protein is important for the function of secretory vesicles and thereby for cytokine secretion.	Secretory vesicles<br>Cytokine release
**ACPP**	*Acid phosphatase protein/prostatic acid phosphatase.* This enzyme colocalizes with the SNAPIN protein (SNAP associated protein), the SNARE (soluble N-ethylmaleimide-sensitive fusion protein attachment protein receptor) complex of proteins and the BLOC-1 (biogenesis of lysosome-related organelles) complex. The SNARE complex is required for vesicle docking and fusion and regulates extracellular mediator release [6].	Vesicle docking<br>Extracellular release<br>Cytokines
**SLC2A3**	*Solute carrier family 2 member 3 (synonym Glut3).* Glut3 is localized at the cell surface but also in a distinct population of intracellular vesicles with high level of	Secretory vesicles

	aminopeptidase B. This enzyme is a marker of a secretory pathway [7]. Furthermore, gene expression studies have shown that SLC2A3 expression is decreased in leukemic blast cells, especially in cells derived from patients with adverse prognosis, and this effect is possibly mediated through altered epigenetic regulation and altered TET activity [8].	Decreased levels associated with adverse prognosis in AML
**STOM**	*Stomatin.* This membrane protein may regulate ion channels and transporters.	Ion transport?
**ATP11B**	*ATPase phospholipid transporting 11B.* P-type ATPases drive uphill transport of ions across membranes. Several subfamilies of P-type ATPases have been identified. One subfamily transports heavy metal ions, such as Cu^{2+} or Cd^{2+}, one of them transporting amphipaths, such as phosphatidylserine. ATP11B mediates chemotherapy resistance in cancer cells [9]. It is a member of the ATP11 subfamily of P4-ATPases, it regulates the membrane distribution of phosphatidylserine in membranes and may thereby influence vesicular transport [10].	Chemotherapy resistance<br>Vesicular transport<br>Ion transport?<br>Membrane structure
**SERPINB6**	Serpin family B member 6. This protein is a member of the serpin (serine proteinase inhibitor) superfamily and ovalbumin (ov)-serpin subfamily.	Protease inhibitor
**ITGAX**	*Integrin subunit alpha x.* This integrin protein combines with the beta 2 chain (ITGB2) to form a leukocyte-specific integrin referred to as inactivated-C3b (iC3b) receptor 4. The dimer is important for phagocytosis of complement coated particles.	Integrin<br>Complement receptor
**NBEAL2**	*Neurobeachin like 2.* The protein is probably involved in the formation of secretory granules that are involved in the extracellular release of cytokines [11].	Secretory granules<br>Cytokine release
	**Platelet Degranulation (9 Proteins)**	
**ASAH1**	*N-acylsphingosine amidohydrolase 1.* This lysosomal enzyme is a member of the acid ceramidase family and catalyzes the degradation of ceramide into sphingosine and free fatty acid. The enzyme may be a potential therapeutic target in AML [12,13].	Lysosome<br>AML
**TGFB1**	*Transforming growth factor beta 1.* This secreted ligand binds its receptor leading to recruitment and activation of SMAD family transcription factors. This encoded protein regulates cell proliferation/differentiation and modulates expression and activation of other growth factors. It seems to have complex and context-dependent effects in AML probably mediated through autocrine and paracrine loops [14–16].	Cytokine
**ACTN1**	*Actinin alpha 1.* Alpha actinins belong to the spectrin gene superfamily that represents a diverse group of cytoskeletal proteins. This non-muscle actin-binding protein is found along microfilament bundles and adherens-type junctions, where it is involved in binding actin to the membrane.	Cytoskeleton
**PPBP**	*Pro-platelet basic protein.* The protein belongs to the CXC chemokine family and stimulates various cellular processes including DNA synthesis, mitosis, glycolysis, intracellular cAMP accumulation, prostaglandin E2 secretion and synthesis of hyaluronic acid and sulfated glycosaminoglycan.	Growth factor<br>Metabolism
**VIM**	*Vimentin.* This type III intermediate filament protein is a part of the cytoskeleton and stabilizes cytoskeletal interactions. This protein is thereby involved in cell attachment, migration, and signaling.	Cytoskeleton
**SRGN**	*Serglycin.* This protein is a hematopoietic cell granule proteoglycan stored in the secretory granules of many hematopoietic cells and is associated with the macromolecular complex of granzymes and perforin, which may serve as a mediator of granule-mediated apoptosis.	Granule-mediated apoptosis
**SPARC**	*Secreted protein acidic and cysteine rich.* This matrix-associated protein has been associated with tumor suppression but has also been correlated with metastasis based on changes to cell shape, which can promote tumor cell invasion.	Matrix protein<br>Tumor suppressor?
**FERMT3**	*Fermitin family member 3.* Kindlins are a small family of proteins that mediate protein-protein interactions involved in integrin activation and thereby have a role in cell adhesion/migration and proliferation. This protein also seems to maintain the membrane skeleton.	Integrins<br>Membrane skeleton
**THBS1**	*Thrombospondin 1.* The protein is a subunit of an adhesive glycoprotein that mediates cell-to-cell and cell-to-matrix interactions. This protein can bind to fibrinogen, fibronectin, laminin, type V collagen and integrins alpha-V/beta-1. It may be involved in carcinogenesis/angiogenesis.	Matrix, adhesion<br>Integrin<br>Angiogenesis
	**Actin Cytoskeleton (9 Proteins)**	
**MYH14**	*Myosin heavy chain 14.* Myosins are actin-based motor proteins and this protein represents a conventional non-muscle myosin. Myosins transport a wide variety of cargo, ranging from vesicles to ribonuclear protein particles. The properties of a given myosin transporter are adapted to move on different actin filament tracks. Transport seems to be controlled by selective recruitment of the myosin to its cargo [17].	Cytoskeleton<br>Intracellular transport
**MYH9**	*Myosin heavy chain 9.* This conventional non-muscle myosin is a myosin IIA heavy chain is probably involved in several important functions, including cytokinesis, cell motility and maintenance of cell shape.	Cytoskeleton<br>Intracellular transport

**MYL6**	*Myosin light chain 6.* Myosin is a hexameric ATPase cellular motor protein. It is composed of two heavy chains, two alkali light chains, and two regulatory light chains. This gene encodes a myosin alkali light chain that is also expressed in non-muscle tissues. Genomic sequences representing several pseudogenes have been described and two transcript variants encoding different isoforms have been identified for this gene.	Cytoskeleton<br>Intracellular transport
**MYL12A**	*Myosin light chain 12A.* This nonsarcomeric myosin regulatory light chain that regulates non-muscle cell contraction. It may also be involved in DNA damage repair.	Cytoskeleton<br>Intracellular transport
**ZYX**	*Zyxin.* Focal adhesions are actin-rich structures that enable cells to adhere to the extracellular matrix and at which protein complexes involved in signal transduction assemble. Zyxin is a zinc-binding phosphoprotein that concentrates at focal adhesions and along the actin cytoskeleton. Its proline-rich domain may interact with SH3 domains of proteins involved in signal transduction pathways while the LIM domains are likely involved in protein-protein binding. It may also modulate the cytoskeletal organization of actin bundles. The protein may be important in leukemogenesis [18].	Cytoskeleton<br>Signaling<br>Adhesion<br>Leukemogenesis?
**ROCK2**	*Rho associated coiled-coil containing protein kinase 2*. It is a serine/threonine kinase that regulates cytokinesis, smooth muscle contraction, the formation of actin stress fibers and focal adhesions, and the activation of the c-fos serum response element. This protein is a target for the small GTPase Rho [19].	Cytokinesis
**TLN1**	*Talin 1.* This cytoskeletal protein has a significant role in the assembly of actin filaments and in spreading and migration of cells. It codistributes with integrins in the cell surface membrane in order to assist in the attachment of adherent cells to extracellular matrix and to other cells. The N-terminus contains elements for localization to cell-extracellular matrix junctions whereas the C-terminus contains binding sites for beta-1-integrin, actin, and vinculin.	Cytoskeleton<br>Integrin<br>Actin
**VCL**	*Vinculin.* Vinculin is a cytoskeletal protein associated with cell-cell and cell-matrix junctions, where it is thought to function as one of several interacting proteins involved in anchoring F-actin to the membrane.	Cytoskeleton<br>Matrix, adhesion
**NFKB1**	*Nuclear factor kappa B subunit 1.* This gene encodes a 105 kD protein, which can undergo cotranslational processing by the 26S proteasome to produce a 50 kD protein. The 105 kD protein is a Rel protein-specific transcription inhibitor and the 50 kD protein is a DNA binding subunit of the NFκB transcription regulator.	Transcription factor

References

1. Aasebo, E.; Berven, F.S.; Bartaula-Brevik, S.; Stokowy, T.; Hovland, R.; Vaudel, M.; Doskeland, S.O.; McCormack, E.; Batth, T.S.; Olsen, J.V., et al. Proteome and Phosphoproteome Changes Associated with Prognosis in Acute Myeloid Leukemia. *Cancers (Basel)* **2020**, *12*, doi:10.3390/cancers12030709.
2. Kitamura, T.; Naganuma, T.; Abe, K.; Nakahara, K.; Ohno, Y.; Kihara, A. Substrate specificity, plasma membrane localization, and lipid modification of the aldehyde dehydrogenase ALDH3B1. *Biochim. Biophys. Acta* **2013**, *1831*, 1395–1401, doi:10.1016/j.bbalip.2013.05.007.
3. Barber, C.N.; Huganir, R.L.; Raben, D.M. Phosphatidic acid-producing enzymes regulating the synaptic vesicle cycle: Role for PLD? *Adv. Biol. Regul.* **2018**, *67*, 141–147, doi:10.1016/j.jbior.2017.09.009.
4. Veit, M.; Koyro, K.I.; Ahrens, B.; Bleibaum, F.; Munz, M.; Rovekamp, H.; Andra, J.; Schreiber, R.; Kunzelmann, K.; Sommer, A., et al. Anoctamin-6 regulates ADAM sheddase function. *Biochim. Biophys. Acta Mol. Cell Res.* **2018**, *1865*, 1598–1610, doi:10.1016/j.bbamcr.2018.08.011.
5. Lokau, J.; Agthe, M.; Flynn, C.M.; Garbers, C. Proteolytic control of Interleukin-11 and Interleukin-6 biology. *Biochim. Biophys. Acta Mol. Cell Res.* **2017**, *1864*, 2105–2117, doi:10.1016/j.bbamcr.2017.06.008.
6. Staley, L.A.; Ebbert, M.T.; Bunker, D.; Bailey, M.; Alzheimer's Disease Neuroimaging, I.; Ridge, P.G.; Goate, A.M.; Kauwe, J.S. Variants in ACPP are associated with cerebrospinal fluid Prostatic Acid Phosphatase levels. *BMC Genomics* **2016**, *17 Suppl 3*, 439, doi:10.1186/s12864-016-2787-y.
7. Balogh, A.; Cadel, S.; Foulon, T.; Picart, R.; Der Garabedian, A.; Rousselet, A.; Tougard, C.; Cohen, P. Aminopeptidase B: A processing enzyme secreted and associated with the plasma membrane of rat pheochromocytoma (PC12) cells. *J. Cell Sci.* **1998**, *111 (Pt. 2)*, 161–169.
8. Liu, J.; Hong, J.; Han, H.; Park, J.; Kim, D.; Park, H.; Ko, M.; Koh, Y.; Shin, D.Y.; Yoon, S.S. Decreased vitamin C uptake mediated by SLC2A3 promotes leukaemia progression and impedes TET2 restoration. *Br. J. Cancer* **2020**, 10.1038/s41416-020-0788-8, doi:10.1038/s41416-020-0788-8.

9. Moreno-Smith, M.; Halder, J.B.; Meltzer, P.S.; Gonda, T.A.; Mangala, L.S.; Rupaimoole, R.; Lu, C.; Nagaraja, A.S.; Gharpure, K.M.; Kang, Y., et al. ATP11B mediates platinum resistance in ovarian cancer. *J. Clin. Invest.* **2018**, *128*, 3199, doi:10.1172/JCI122301.
10. Wang, J.; Li, W.; Zhou, F.; Feng, R.; Wang, F.; Zhang, S.; Li, J.; Li, Q.; Wang, Y.; Xie, J., et al. ATP11B deficiency leads to impairment of hippocampal synaptic plasticity. *J. Mol. Cell Biol.* **2019**, *11*, 688–702, doi:10.1093/jmcb/mjz042.
11. Moqbel, R.; Coughlin, J.J. Differential secretion of cytokines. *Sci. STKE* **2006**, *2006*, pe26, doi:10.1126/stke.3382006pe26.
12. Tan, S.F.; Liu, X.; Fox, T.E.; Barth, B.M.; Sharma, A.; Turner, S.D.; Awwad, A.; Dewey, A.; Doi, K.; Spitzer, B., et al. Acid ceramidase is upregulated in AML and represents a novel therapeutic target. *Oncotarget* **2016**, *7*, 83208–83222, doi:10.18632/oncotarget.13079.
13. Pearson, J.M.; Tan, S.F.; Sharma, A.; Annageldiyev, C.; Fox, T.E.; Abad, J.L.; Fabrias, G.; Desai, D.; Amin, S.; Wang, H.G., et al. Ceramide Analogue SACLAC Modulates Sphingolipid Levels and MCL-1 Splicing to Induce Apoptosis in Acute Myeloid Leukemia. *Mol. Cancer Res.* **2020**, *18*, 352–363, doi:10.1158/1541-7786.MCR-19-0619.
14. Bruserud, O. Acute myelogenous leukemia blasts as accessory cells during T lymphocyte activation: Possible implications for future therapeutic strategies. *Leukemia* **1999**, *13*, 1175–1187, doi:10.1038/sj.leu.2401452.
15. Geyh, S.; Rodriguez-Paredes, M.; Jager, P.; Koch, A.; Bormann, F.; Gutekunst, J.; Zilkens, C.; Germing, U.; Kobbe, G.; Lyko, F., et al. Transforming growth factor beta1-mediated functional inhibition of mesenchymal stromal cells in myelodysplastic syndromes and acute myeloid leukemia. *Haematologica* **2018**, *103*, 1462–1471, doi:10.3324/haematol.2017.186734.
16. Ruscetti, F.W.; Akel, S.; Bartelmez, S.H. Autocrine transforming growth factor-beta regulation of hematopoiesis: Many outcomes that depend on the context. *Oncogene* **2005**, *24*, 5751–5763, doi:10.1038/sj.onc.1208921.
17. Titus, M.A. Myosin-Driven Intracellular Transport. *Cold Spring Harb. Perspect. Biol.* **2018**, *10*, doi:10.1101/cshperspect.a021972.
18. Hervy, M.; Hoffman, L.; Beckerle, M.C. From the membrane to the nucleus and back again: Bifunctional focal adhesion proteins. *Curr. Opin. Cell Biol.* **2006**, *18*, 524-532, doi:10.1016/j.ceb.2006.08.006.
19. Haga, R.B.; Garg, R.; Collu, F.; Borda D'Agua, B.; Menendez, S.T.; Colomba, A.; Fraternali, F.; Ridley, A.J. RhoBTB1 interacts with ROCKs and inhibits invasion. *Biochem. J.* **2019**, *476*, 2499–2514, doi:10.1042/BCJ20190203.

.

© 2020 by the authors. Licensee MDPI, Basel, Switzerland. This article is an open access article distributed under the terms and conditions of the Creative Commons Attribution (CC BY) license (http://creativecommons.org/licenses/by/4.0/).
